# Influence of hypertension classification on hypertensive intracerebral hemorrhage location

**DOI:** 10.1111/jch.14367

**Published:** 2021-10-05

**Authors:** Jun Shen, FengBao Guo, Peng Yang, Feng Xu

**Affiliations:** ^1^ Department of Emergency Medicine The First Affiliated Hospital of Soochow University Suzhou China

**Keywords:** hypertension classification, lobar, non‐lobar, prognosis

## Abstract

The authors sought to explore whether hypertension classification was risk factor for lobar and non‐lobar hypertensive intracerebral hemorrhage (HICH) and the prognosis in patients with hematoma. This retrospective cohort study was conducted on HICH patients admitted at the First Affiliated Hospital of Soochow University. Observations with first‐ever intracerebral hemorrhage (ICH) were recruited. The authors divided the brain image into three groups according to the location of ICH to predict whether there were significant differences between lobar and non‐lobar ICH. A Mann‐Whitney U test was used and this retrospective trial also compared the operation and mortality rates. Our cohort included 209 patients (73.7% male; median age:60.5±16.7). The overall incidence of lobar HICH was less than non‐lobar HICH (24.4% vs. 68.4%), 7.2% cases of mixed HICH was included in this analysis. In a Mann‐Whitney U test analyze, it indicated that there were significant differences in hypertension classification between lobar and non‐lobar HICH (Z = ‐3.3, *p*<.05). And the percentage of hematoma in lobar areas with relatively slightly high blood pressure (BP) (high normal and grade 1 hypertension) accounts for 52.9% versus 30.1% in non‐lobar areas. The increasing trends of the prevalent rate of lobar ICH with BP rising were not remarkable. The non‐lobar HICH showed a sharper increase in the condition of grade 3 hypertension compared with lobar HICH. During the period of research, the fatality of lobar hemorrhage was 2.9% versus 7.7% (non‐lobar). Besides, the fatality incidence of HICH with relatively slightly high BP (high normal and grade 1 hypertension) was lower than poorly controlled hypertensive patients (grade 2 and grade 3 hypertension). (8.0% vs. 15.7%). The increase of hypertension classification will aggravate the occurrence of non‐lobar ICH and positively corrected with BP, but not in lobar areas. It is essential to understand the distinction influence of hypertension classification between lobar and non‐lobar ICH.

## 1 INTRODUCTION

The annual number of deaths owing to cardiac‐cerebral vascular disease increased sharply in recent years,^1^, ^2^, ^3^, ^4^ it may cause a considerable economic burden on the medical and health care system.^5^ Besides, impressive younger trend disease had been observed.^6^, ^7^ In the representative subcategories, the occurrence of ICH attracted more attention and increasing research interest than ever.^8^ ICH is known to have a variety of causes, including hypertension, vascular malformations, cerebral amyloid angiopathy (CAA), brain tumors, and trauma.^9^, ^10^, ^11^ Hypertension‐related hemorrhage was the most common forms.^12^, ^13^ which may lead to acute clinical symptoms. In patients with HICH, basal ganglia was the most common site, followed by thalamus and lobar hemorrhage.^7^ In pathology studies, raised blood pressure (BP) may lead to rupture of both lobar and non‐lobar small arteries.^14^ Hypertension associated lesions affecting small vessels in the deep perforating arteries are to a large extent similar to those that affect leptomeningeal and cortical vessels. The causes and prognosis of HICH may be determined by anatomical location.^15^ However, under varies BP conditions, the tolerance and sensitivity of the small vessels in the brain are different. This indirectly leads to the hypertension classification affecting the location and prognosis of ICH. Identification of hypertension classification of HICH is essential because it might imply the hemorrhage location to some extent. The location of ICH is an important cause of the prognosis of disease. The aim of the research is to provided risk factors to enable understanding the relationship between hypertension classification and hematoma location, and encourage the measures of lowing BP to protect this high‐risk patient group.

## 2 METHODS

### 2.1 Study design and participants

We did a retrospective research of HICH in the First Affiliated Hospital of Soochow University from November 30, 2019 to October 31, 2020. We identified incident HICH cases using multiple overlapping sources of medical data system. All suspected patients with history of hypertension and first attack were assessed by a physician through performing Computed Tomography/Computed Tomography Angiography (CT/CTA) as soon as possible after admission. Two‐hundred and nine patients were enrolled and their clinical and image data were reviewed retrospectively. We included adults patients aged from 25 to 97 years, including 154 males and 55 females. The inclusion criteria were the followings: (1) first‐ever ICH onset; (2)hypertension with a history or moment of documented BP elevations of greater than 90 mm Hg diastolic or 140 mm Hg systolic; (3) make a definite diagnosis by CT or MRI (Magnetic Resonance Imaging); (4)obvious clinical symptoms, such as intracranial hypertension, hemiplegia and so on. Exclusion criteria were carried out when the patient met the followings: (1)arteriovenous malformation (AVM) or other vascular abnormalities; (2) recurrent ICH; (3) hemorrhages with identified secondary causes, such as ICH secondary to trauma; (4) hemorrhagic transformation of an ischemic stroke; (5) CAA; (6) space‐occupying lesions.

### 2.2 Definition of lobar and non‐lobar HICH

We divided the site of HICH into three categories. Firstly, risk factors were accessed according to lobar and non‐lobar location. Lobar HICH location included several cerebral lobe sub‐regions (frontal lobe, temporal lobe, parietal lobe, and occipital lobe). Non‐lobar HICH location included two areas. Infratentorial ICH represented that patients had hemorrhage in the brain stem or cerebellum. And a supratentorial deep HICH which located in basal ganglia, internal or external capsule or thalamus was identified. Intraventricular hemorrhage was excluded as a consequence of hematoma expansion, which may lead to difficult measure of volume.^16^, ^17^ Besides, all mixed ICHs were supposed to be the same category.^18^

### 2.3 Treatment and prognosis information

We collected clinical information by reviewing hospital records. The records and radiographic data were analyzed in terms of patient age, sex, admission time, the presence of hypertension, neurological symptoms and signs, CT features, location of hematomas, and outcome. We reviewed the hospital records to search whether the patients performed surgical treatment. Surgical operations mostly because emergency surgery was required for a life‐threatening hematoma. The influence of hypertension classification on the site of ICH was reflected by comparing the operation rate of patients with lobar or non‐lobar. Moreover, we are also concerned about the prognosis including survival or death. Correlation ratios can reflect some variation.

### 2.4 Regulatory approval

Patient's data was anonymized and de‐identified and the protocol of this research was approved by the institutional ethics committee of each collaborating center of our hospital due to the retrospective nature of the data. This study conformed to the principles of the Declaration of Helsinki.

### 2.5 Statistical analysis

After completing recruitment to this retrospective study, we used the sample size of 209 and restricted models to variables. Categorical variables were described as frequencies and percentages. Baseline characteristics were summarized by means and standard deviations (SD) for normally distributed variables, medians and interquartile ranges (IQR) for skewed continuous variables. A Mann‐Whitney U test was used to investigate the differences related to the hemorrhage location. A general significance level of *p* < .05 was applied. Statistical analysis was performed by the means of SPSS 24 statistical software (IBM SPSS Statistics).

## 3 RESULTS

**FIGURE 1:**
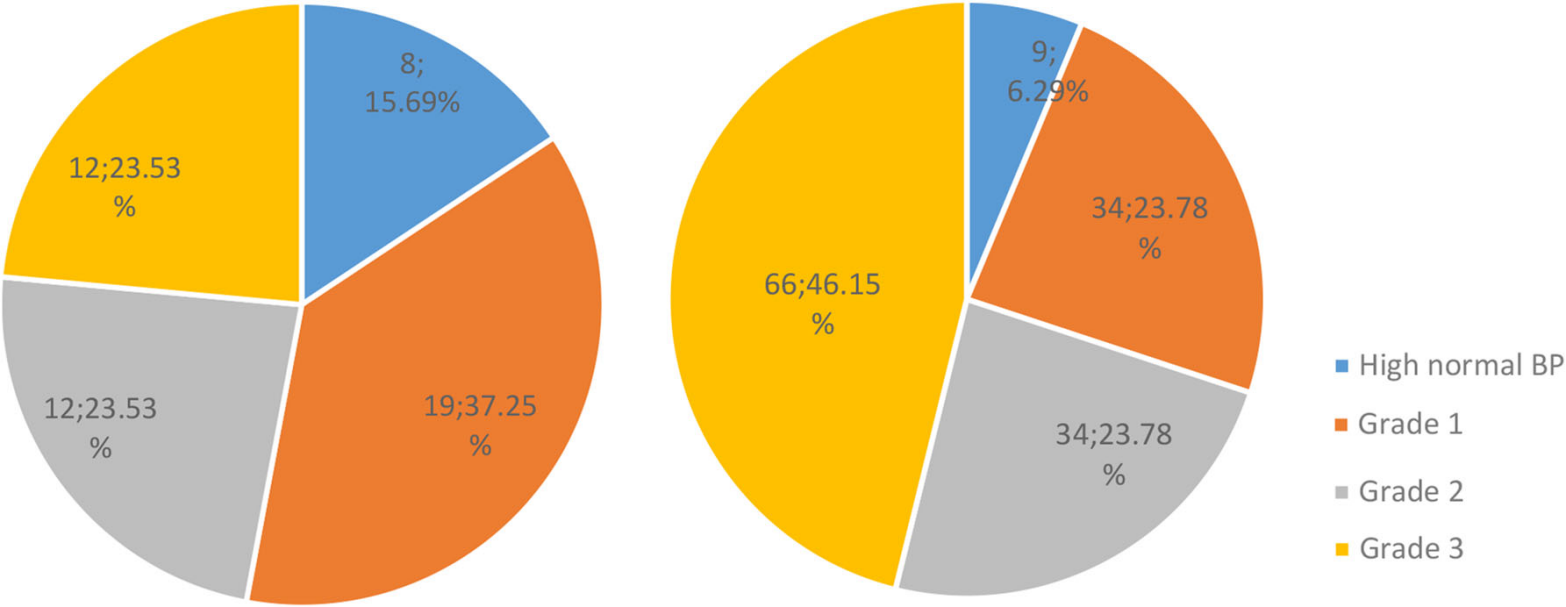
Left pie diagram represented a scale model of lobar HICH and the right represented non‐lobar HICH. Both high normal and grade 1 hypertension showed an increase percentage in lobar areas than non‐lobar (52.9% vs. 30.1%). And the non‐lobar HICH showed an increase with the blood pressure level increasing

We recruit sample size (*n* = 209, 154 [73.7%] male, 55 [26.3%] female) from the patients admission into the First Affiliated Hospital of Soochow University. The median age of all patients were 60.5±16.7. The mean BP readings measured immediately after admission were 168.7±26.1 mm Hg/97.4±18.5 mm Hg. The three location of HICH were lobar (51, 24.4%), non‐lobar (143, 68.4%) (infratentorial [22], supratentorial [121]), and mixed (15, 7.2%), respectively. Those patients presented differed significantly in sex, grade 3 hypertension, volume and mean number of readings of BPs (*p*<.05), but not in age, history of diabetes, smoking, drinking, hyperlipidemia and other classification of hypertension (*p*>.05). (table 1) Demographic and clinical baseline characteristics of all patients are summarized in Table 2. In a Mann‐Whitney U test, we can conclude that there were significant differences in hypertension classification between lobar and non‐lobar HICH (Z = ‐3.3, *p*<.05). And the percentage of hematoma in lobar areas with relatively slightly high BP (high normal and grade 1 hypertension) accounts for 52.9% versus 30.1% in non‐lobar areas. The increasing trends of the prevalent rate of lobar ICH with BP rising were not remarkable. The prevalence of non‐lobar ICH increased stepwise from 23.8% in the grade 2 hypertension group to 46.1% in the grade 3 hypertension group, and showed a sharper increase in the condition of grade 3 hypertension than lobar HICH.(46.1% vs. 23.5%) (Figure 1) Hypertension classification was associated with a higher prevalence of non‐lobar HICH compared with lobar areas. Across the entire operation patients (*n* = 84), lobar hemorrhage accounted for 10.5% (*n* = 22), and the non‐lobar hemorrhage accounted for 24.4% (*n* = 51), and mixed were 5.3% (*n* = 11) (Table 2). People with non‐lobar hemorrhage were more likely to achieve an operative treatment than lobar hemorrhage. (24.4% vs. 10.5%) Besides, the fatality of lobar and non‐lobar accounted for 2.9% and 7.7%, respectively. On the whole, the fatality incidence of HICH with relatively slightly high BP (high normal and grade 1 hypertension) was lower than poorly controlled hypertensive patients (grade 2 and grade 3 hypertension) (8.0% vs. 15.7%) (Table 2).

**TABLE 1:** Characteristics of HICH associated with location

	Lobar HICH (*N* = 51)	Non‐lobar HICH (*N* = 143)	*p* value
Age (years)	66 (64.7 ± 16.6)	57 (59.3 ± 16.8)	>.01
Sex			<.01
Male	26	116	
Female	25	27	
History of diabetes			
Yes, *n*(%)	6 (11.8)	24 (16.8)	>.01
Smoking history			
Yes, *n*(%)	3 (5.9)	21 (14.7)	>.01
History of drinking			
Yes, *n*(%)	4 (7.8)	20 (14.0)	>.01
History of hyperlipidemia			
Yes, n(%)	9 (17.6)	35 (24.5)	>.01
Blood pressure classification			
high normal BP (%)	8 (15.7)	9 (6.3)	>.01
grade 1 hypertension (%)	19 (37.3)	34 (23.8)	>.01
grade 2 hypertension (%)	12 (23.5)	34 (23.8)	>.01
grade 3 hypertension (%)	12 (23.5)	66 (46.1)	<.01
Volume, median (IQR)	40.7 (23.1, 62.6)	16.4 (6.4, 36.9)	<.01

HICH indicated hypertension intracerebral hemorrhage.

BP indicated blood pressure.

Continuous variables failing to conform to normality were thus expressed as median (inter quartile range, IQR).

**TABLE 2:** Baseline characteristics of HICH

Characteristics	Variables 1	Variables 2	Number of cases
Sex (%)	Male		154 (73.7)
	Female		55 (26.3)
Age (years)			60.5 ± 16.7
Location of hemorrhage (%)	Lobar		51 (24.4)
	Non‐lobar		143 (68.4)
		Supratentorial	121 (57.9)
		Infratentorial	22 (10.5)
	Mixed		15 (7.2)
Volume, median (IQR)			24.3 (8.6, 46.3)
Operation	Yes		84 (40.2)
		Lobar	22 (10.5)
		Non‐lobar	51 (24.4)
		Mixed	11 (5.3)
Outcome	Cured or improved		182 (87.1)
	Not recovered or death		27 (12.9)
		Lobar	6 (2.9)
		Non‐lobar	16 (7.7)
		Mixed	5 (2.4)
		High normal and grade 1	6 (8.0)
		Grade 2 and grade 3	21 (15.7)
Hospitalization time (day)			20.4 ± 16.8

Mixed indicated non‐lobar and lobar ICH.

HICH indicated hypertension intracerebral hemorrhage.

## 4 DISCUSSION

From a pathological point of view, hypertension can cause arteriolosclerosis, an accumulation of degenerative material in the tunica intima (inner layer) of artery walls.^19^ Subsequently, the form of the disease are characterized by fibrinoid necrosis, lipohyalinosis, microatheroma, and microaneurysms.^20^, ^21^ Hypertension‐related mechanisms have been described both in deep perforating intracranial arteries and leptomeningeal and cortical vessels. Stroke patients with BP above this threshold, it may be going to have same effect on lobar and non‐lobar, respectively,^22^ although hypertension is less often a predisposing factor in lobar ICH.^9^, ^23^, ^24^ Our investigation suggested that the increase of hypertension classification will aggravate the number of non‐lobar ICH compared with lobar ICH. Vessels in different anatomical location might be at least partly affected by hypertension to a varying degree. The causes, risk factors and prognosis of spontaneous ICH are partly determined by classification of hypertension. Influence of hypertension classification on hemorrhage location was accessed according to lobar and non‐lobar ICH.^25^ Some possibilities have to be considered to explain the influence of hypertension classification: anatomical structure of vessels, blood vessel sensitivity to hemodynamics and autoregulatory vasodilatation of changes in BP.

According to the relevant vascular and brain anatomy, the lobar category includes individual cerebral lobe sub‐regions. Non‐lobar regions includes sub‐regions of deep and infratentorial areas. More details optional anatomical regions including the basal ganglia, thalamus, brainstem, and cerebellum are acknowledged.^15^ Leptomeningeal and cortical vessels penetrate the brain cortex superficially, supplying the lobar locations. The deep perforating arteries are terminal vessels located deep in the ventral brain area, which are emitted directly from the trunk vessels. Both superficial and deep perforators have very limited collateral connections with adjacent small vessels,^26^ and the medial muscle cells in the small arteries are less than in the arteries of similar diameter in other parts of the body.^27^ These conditions were thought to be the main cause of ICH. The perforating (or deep perforating) arteries, arising from the arterial circle of Willis or from its immediate branches, especially the lenticulostriate artery, exclusively supplied by perforating end arteries originating from the M1 segment of the middle cerebral artery.^28^, ^29^ It comes out vertically at the bifurcations, once the BP increases suddenly, the impact of blood flow will enhance in the anatomical vertical position,^30^ if the BP reaches the points rupture of vessel wall, it may tend to bleeding, such as basal ganglia, the most common site of bleeding. Superficially, although they were terminal vessels stemming from the subarachnoid circulation, the arterioles have branches from the external carotid artery(ECA), and both cortical and subcortical structures are adequately supplied by the posterior cerebral artery,^20^, ^31^, ^32^ forming an anastomotic network on the surface of the hemispheres.^28^ Leptomeningeal collateral flow is a very important and mechanism of perfusion compensation.^33^ Once the BP is elevated, it can buffer some of the pressure, reducing the effect of BP.^34^ And it could explain the reduced ICH risk in lobar areas. The buffer capacity of non‐lobar areas was weaker than lobar areas when the BP remained at the same level. And if the BP exceeded the buffer threshold, the number of ICH increased significantly in non‐lobar areas with the increased of BP level. Lobar HICH might simply be less affected by hypertension than non‐lobar areas.

As we all known, perforating arteries are more proximal to the large arteries, and may more strongly reflect the changes in systemic BP than cortical arteries.^32^ Elevation of systemic BP and vasoconstriction during acute stroke.^35^, ^36^ Active adjustments upon cerebral blood perfusion in vascular muscle tone resulting from changes in intravascular and transmural pressure.^37^ This effect may be a protective mechanism that attenuates changes in pressure in thin‐walled intracranial blood vessels. And the smallest pial arterioles did not change size over a wide range of arterial BP.^38^ Besides, it had revealed that pial arteries on the cortical surface are generally equipped with two to three muscle layers, whereas penetrating arteries have only one to two smooth muscle cells per circumference.^36^ The functioning of autoregulation of deep perforating intracranial arteries are more sensitive and pronounced than that of leptomeningeal and cortical vessels.^32^ Combined with the above conditions, blood vessels in non‐lobar areas might be sensitive to hemodynamics, which are more prone to suffer bleeding. The HICH location may vary on hypertensive classification because of the sensitivity to BP are different in varies parts. Based on our research, the incidence of non‐lobar ICH had close links with hypertension classification, especially in the condition of grade 3 hypertension. Comparatively speaking, the functioning of autoregulation in lobar arteries was weaker, resulting in unremarkable increasing trends in lobar ICH.

Typical artery atheromatous plaques may exist in all intracranial arteries, but the mechanisms of arteriolar degeneration varied depending on vessel histological structure and function in various locations. Earlier studies had shown that deep perforating intracranial arteries were larger in diameter than leptomeningeal and cortical vessels,^39^ and the atherosclerotic process affects those arteries with 200 μm or more in diameter.^40^ In addition, the focal thickening of the basement membrane (BM) of cerebral arteries appears to be more obvious at the branching sites.^41^ When the patients were at risk for hypertension, a rupture of a perforating artery in non‐lobar areas may be more common, especially at the branching sites. The hemorrhage occurred at large arteries in non‐lobar areas were impacted more by hypertension classification than in lobar areas. Based on the above reasons, the changes of the vascular wall at various locations can explain the associated between hypertension and ICH location. Interestingly, patients with cerebral infarction and HICH may indicate vascular injury caused by atherothrombosis and high BP, respectively. Paradoxically, atherosclerotic plaque may appear different pattern of manifestation in individuals. The immediate rupture of the intracerebral microaneurysm may cause bleeding.^27^ Hypotension, slow blood flow and increased platelet cohesion may increase vascular resistance, and eventually developed thrombosis. The type of ischemic cerebrovascular disease caused by the atherosclerosis is mainly large cerebral infarction.^42^, ^43^ Cerebral infarction might be affected by hypertension classification as same as ICH, but this needs further investigation.

High normal and grade 1 hypertension at our study were associated with a significantly higher risk of ICH in lobar areas compared with non‐lobar areas. However, the increasing trends of the prevalent rate of lobar ICH with BP rising were not remarkable. Many studies suggested that cardiovascular diseases (CVDs) could start even with relatively slightly high BP in the general population.^44^, ^45^ The association between BP category and lobar ICH was observed in our study. We need to be aware that the potential pathophysiological significance of relatively slightly high BP, even in low‐risk populations.^46^ Few studies have assessed to what extent BP classification was associated with ICH location. Whether hypertension classification had an effect on lobar hemorrhage cannot be found in our study, a further study is needed. Besides, we found that a considerable proportion of the ICH patients suffered with relatively low level of BP in our study. In fact, ICH will cause compression of brain tissue and nerve pathway injury, which especially in pivotal region for maintaining basal BP and sympathetic tone, causing lower BP.^47^ Falls in BP was associated with neurological deterioration which induced impaired cerebral blood flow autoregulation, cerebral hypoperfusion and insufficient circulating blood volume.^42^ In addition, many complications can directly lead to lower BP, such as brain hernia, digestive tract bleeding, heart failure.

In our study, it had be shown that lobar HICH had greater average volume than non‐lobar HICH, it was consistent with many studies.^48^, ^49^, ^50^ The lobar areas provided more space for the hemorrhage and white‐matter hyperintensities in different parts had an effect on the volume of hemorrhage.^51^ The non‐lobar areas represented a passing fortress crucial for patients, thus deep bleeding may increases the risks of death or disability and result in poor motor function.^52^ Whereas lobar HICH neared cortex and cortical‐subcortical junction, and were less likely to extend into the ventricular system. Intraventricular volume has been postulated as an important predictor for worsening prognosis.^53^, ^54^, ^55^ Two counterbalancing cancel each other out in some degree.^56^ So lobar HICH was more likely to be weakened in common operative assessments and identified less hematoma expansion, leading to have a relatively benign prognosis.^57^, ^58^ However, some researchers also concluded that lobar ICH and deep cerebral ICH had similar fatality rates, contrary to some older reports.^56^ It may depend on the bias of the study patients we selected.

ICH was widely considered to be fatal and the most disabling stroke subtype. A majority of the cases who died as a direct consequence of the neurological deterioration, and the operation depends on stroke severity. Lobar ICH had been associated with a better outcome in some studys.^59^ We found that patients with hemorrhage located lobar areas were not only more likely to reduce the risk of operation but also had long‐term survival. The fatality and operative rate in the present study was consistent with other large studies.^52^ The fatality incidence of HICH with relatively slightly high BP was lower than poorly controlled, it could illustrate that hypertension classification also had an impact on the prognosis of ICH.

## 5 LIMITATIONS

Our study had some limitations. Firstly, we only enrolled a small sample of patients in a single center, and thus unrepresentative. There is likely still some residual confounding of our estimates. For example, other factors may also play a role in HICH, including antithrombotic drug and anticoagulant drug use, but we did not obtain the data of BP and the usage of antiplatelet and/or anticoagulation therapy. Secondly, some patients were excluded for poor image quality and this may lead to bias.^33^ Limited from research means, the index BP measurements that should be determined a few days before the stroke attack were not recorded, and we did not know how well the patient's BP was controlled before the hemorrhage. Besides, we did not follow up the patients’ prognosis regularly, it may expose the limitations of research midpoints. A definition of hypertension based on raised BP before stroke may be have a bias, because when the patients suffered with ICH, the BP was often raised. The BP does not reflect that before stroke.

## 6 CONCLUSIONS

Lobar HICH might simply be less affected by hypertension than non‐lobar areas, because leptomeningeal and cortical vessels might simply be less affected by hypertension than deep perforating arteries.^14^ The number of HICH increased significantly in non‐lobar areas with the increased of BP level. There was a trend towards improved outcome in the lobar intracerebral hemorrhage than in the deep locations.^23^ In any cases, more attention and target efforts should be implemented to understand the hemorrhage location caused by hypertension, especially in relationship with hypertensive degree.^20^ Actively controlling BP is the essence of preventing ICH. The hypertension classification might inform prognostic and therapeutic decisions that depend on the identification of hemorrhage locations.

## CONFLICTS OF INTEREST

Authors have no financial or other conflicts of interest related to this submission.

## AUTHOR CONTRIBUTIONS

Jun Shen and Feng Xu conceived the project idea and collected the clinical data. FengBao Guo and Peng Yang provided critical suggestions for this design and provided the imaging analysis. Jun Shen drafted the manuscript and all authors revised it critically for important intellectual content. Feng Xu supervised the project. All authors made substantial contributions to the interpretation of data. All authors approved the final version of the manuscript.

## Supporting information

Supporting information
