# Supplementary material for: Influence of hypertension classification on hypertensive intracerebral hemorrhage location
Source: J Clin Hypertens (Greenwich). 2021 Oct 5;23(11):1992–9. doi: 10.1111/jch.14367 (PMC8630601; doi:10.1111/jch.14367)
Supplement: Supplementary file 1 — Supporting information [file JCH-23-1992-s001.pdf]

苏州大学附属第一医院医学伦理委员会

回顾性研究便捷审核表

审核编号：（2021）伦研批第 123 号

研究项目名称	高血压分级对脑出血部位的影响		
研究类别	回顾性研究		
项目来源	无		
项目科室	急诊科	项目负责人	徐峰
主要研究者	沈军，徐峰，杨鹏		
便捷审查内容	从病例数据库中导出 2019 年 11 月 30 日-2020 年 10 月 31 日的高血压脑出血患者临床相关数据并进行统计分析，探讨高血压分级是否是脑叶及非脑叶型高血压出血患者的危险因素。所有数据将经过去患者个人隐私信息严格脱敏。		
科研立项审查意见	同意立项。<br>签名：[illegible] 2021 年 5月 12日		
便捷审查意见	经伦理委员会审核，该申报项目为回顾性临床研究，研究方案设计科学，符合伦理原则。		
伦理委员会审核委员签字	委员：[illegible] 苏州大学附属第一医院医学伦理委员会（盖章）<br>2021 年 5 月 12日		

附件 1：文章或研究方案

附件 2：科研诚信承诺书

地址：江苏省苏州市平海路 899 号 No.899 Pinghai Street,Suzhou,Jiangsu,China,215006
电话&传真：0512-67972639,67972743 E-mail：sdfyec@163.com
